# Single-Center Experience in Cases With Rib Fractures: When To Be Alert?

**DOI:** 10.7759/cureus.50060

**Published:** 2023-12-06

**Authors:** Omer Giray Intepe, Ayten Guner Akbiyik

**Affiliations:** 1 Thoracic Surgery, Republic of Türkiye Ministry of Health, Ali Osman Sonmez Oncology Hospital, Bursa, TUR; 2 Thoracic Surgery, Republic of Türkiye Ministry of Health, Goztepe Prof. Dr. Suleyman Yalcin City Hospital, Istanbul, TUR

**Keywords:** respiratory insufficiency, hemothorax, pneumothorax, trauma, rib fractures

## Abstract

Aim: The aim of this study was to evaluate rib fracture-related complications in blunt chest traumas.

Methodology: The study included a cohort of 132 male and 42 female patients, aged between 22 and 89 years, all diagnosed with rib fractures subsequent to blunt chest trauma. The data collection period extended from November 2017 to November 2019. Pulmonary complications, including pneumothorax, hemothorax, pulmonary contusion, flail chest, and the need for mechanical ventilator support, were retrospectively evaluated based on age, gender, trauma history, bilateral fractures, the number of fractured ribs, and concomitant traumas in other systems. Patients with one or two fractured ribs were included in Group 1, while those with three or more rib fractures were in Goup 2.

Results: Patients in Group 2 (n=82) had a significantly higher mean age and complication rate compared to patients in Group 1 (56.24 vs. 51.08; p: 0.033; p=0.000). Falls from height were the most common trauma history. The most frequently broken ribs were the fifth right (n=35) and the ninth right ribs (n=35), followed by the seventh right (n=33) and the seventh left rib (n=32) in order. Pneumothorax was diagnosed in 60 patients (34.4%), hemothorax in 48 patients (27.5%), and pulmonary contusion in 22 patients (12.6%). Seven patients had a flail chest (4.0%) and four required mechanical ventilation support. The number of male patients was significantly higher (p=0.000). Motor vehicle accidents were most correlated with complications in trauma history (p=0.002). Elderly age, bilateral fractures, three or more fractured ribs, and the mechanism of trauma were significantly correlated with complications (p < 0.05). The mortality rate was 0%.

Conclusion: Three or more fractured ribs, bilateral fractures, and high-energy traumas are important risk factors, particularly in the elderly population. For patients meeting these criteria, hospitalization and careful observation are recommended.

## Introduction

Blunt chest trauma is more common than penetrating trauma, particularly in high-energy traumas, and can result in rib fractures [1,2]. Bulger et al. noted that, annually, more than 350,000 patients in the United States experience traumas related to rib fractures [3]. Rib fractures can have significant negative effects on respiratory physiology by disrupting chest wall stability and causing damage to vascular structures and lung parenchyma. These adverse effects contribute to high incidences of morbidity and mortality, especially in the elderly population [3-5]. Based on clinical experiences, this study aims to identify alert criteria that can indicate the severity of the situation in cases with rib fractures.

## Materials and methods

The study included a cohort of 132 male and 42 female patients, aged between 22 and 89, all diagnosed with rib fractures subsequent to blunt chest trauma. The data collection period extended from November 2017 to November 2019. The study was conducted in the Emergency Department and Thoracic Surgery Clinic, Kirklareli City Hospital, Türkiye, a hospital that is located in a city with a population of approximately 365,000, with a bed capacity of 275, and served by one thoracic surgeon for the entire city. The study adheres to the principles of the Declaration of Helsinki. The Istanbul Medeniyet University Ethics Committee approved this retrospective study.

Pulmonary complications, including pneumothorax, hemothorax, hemopneumothorax, pulmonary contusion, flail chest, and the need for mechanical ventilator support, were determined based on current data. These complications were retrospectively evaluated by age, gender, trauma history, bilateral fractures, the number of fractured ribs, and other concomitant system traumas.

All cases were hospitalized and carefully monitored. Patients with extrathoracic pathologies were also followed up by related specialists. Mechanical ventilation support was administered in the intensive care unit to patients who needed it. Despite intensive respiratory physiotherapy, in some cases with oxygen desaturation due to remaining secretions in the bronchia, bronchial toilet was performed with fiberoptic bronchoscopy. Intravenous analgesic treatment was provided to patients during their hospital stay. For patients with continuous pain not relieved by intravenous treatment, intercostal nerve blocks were performed. Both emergent and elective surgical intervention algorithms were followed for each patient.

Patients were divided into two groups according to the number of fractured ribs: Patients with one or two fractured ribs were included in Group 1, while those with three or more rib fractures were in Group 2. Regional zones, consisting of an equal number of ribs, were determined on the chest wall: Upper zone (one to four ribs), middle zone (five to eight ribs), and lower zone (9-12 ribs). The number of fractured ribs in each zone was noted. The current recorded database was compatible with the Injury Severity Score (ISS) [6] and Chest Trauma Score (CTS) [7]. These scoring systems were utilized to describe the severity of cases.

SPSS for Windows, Version 16.0. (Released 2007; SPSS Inc., Chicago, United States). Pearson’s Chi-Square Test was performed to describe the relationship between pulmonary complications and age, gender, number of fractures, trauma localization, and the mechanism of trauma. Statistical significance was defined as a two-tailed p-value less than 0.05. The linear regression test was chosen to identify independent risk factors that affect ISS [6] and CTS [7]. The Independent samples t test was selected to reveal the difference between the mean values of ages of Group 1 and Group 2. Mean values of the numbers of fractured ribs in these two groups were also analyzed by the Mann-Whitney U test. To determine if any location on the chest wall was injured more than other parts, the sum of rib fractures in each region (upper, middle, and lower zones on both sides) was counted and analyzed by the Kruskal-Wallis test.

## Results

In the study, there were 132 males (75.9%) and 42 females (24.1%), with an age range of 22-89 years and a mean age of 53.51. Pulmonary complications were detected in 76 cases (43.7%), while the remaining 98 cases (56.3%) were without complications. The group with fewer than 3 rib fractures comprised 92 cases (52.8%), and 82 cases (47.2%) had three or more rib fractures. Of the total cases, 132 (75.9%) were under 65 years old, while the remaining 42 cases (24.1%) were 65 years old or older.

The mechanisms of trauma included falls from <2 meters in 78 cases (44.8%), motor vehicle accidents in 54 cases (31.0%), falls from ≥2 meters in 19 cases (10.9%), assault in six cases (3.4%), crushing under massive objects in six cases (3.4%), being hit by a blunt object in four cases (2.3%), livestock-related traumas in three cases (1.7%), and other blunt traumas, consisting of chronic cough in two cases, rib fracture while lifting a heavy object in one case, and crushing during a massage session in one case, totaling four cases (2.3%).

Seventeen cases had extrathoracic system pathologies concomitantly. These multiple trauma cases included vertebral fractures (n=5), head injuries (n=7), other upper and lower limb fractures (n=7), and intra-abdominal organ pathologies (n=4). Fourteen out of the 17 had pulmonary complications (p=0.008), and 10 of these 17 cases had a history of motor vehicle accidents. Additionally, in this database, five cases had sternum fractures, 11 had clavicle fractures, and nine had scapula fractures.

The most frequently broken ribs were the fifth right (n=35) and the ninth right ribs (n=35), followed by the seventh right rib (n=33) and the seventh left rib (n=32) (Figure 1). There was no significant difference in the distribution of fractured ribs on the left and right sides (left-sided fractures n: 92 (49.7%) vs. right-sided fractures n: 95 (50.3%); p=0.770). However, when bilateral cases were considered separately (82 cases had right-sided rib fractures (47.1%), 79 cases had left-sided rib fractures (45.4%), and 13 cases had bilateral (7.5%)), the distribution of trauma localization was significantly different (p=0.001), and the location of trauma was significantly related to complications (p=0.001). The number of fractured ribs in each case and the corresponding complication status are documented and presented in Table 1.

**Table 1:** Numbers of fractured ribs in each case and complication status

Number of fractured ribs of patients	Number of patients without complication	Number of patients with complication	Total
1	51	9	60
2	23	9	32
3	12	18	30
4	12	15	27
5	0	12	12
6	0	2	2
7	0	5	5
8	0	1	1
10	0	1	1
11	0	1	1
12	0	1	1
13	0	1	1
14	0	2	2
Total	98	76	174

The Kruskal-Wallis Test was conducted to assess which zone of the chest wall was most affected based on the number of fractured ribs in each zone. The test results indicated a significant difference (p=0.002). Binary groups were established to perform the Mann-Whitney U test in each binary group (Test 1: upper vs. middle; Test 2: middle vs. lower; Test 3: upper vs. lower zone) to identify the significantly affected region. The middle zone was identified as the most affected region (Test 1: p= 0.001; Test 2: p= 0.001; Test 3: p>0.05). 

The data on complications and surgical interventions are detailed in Table 2. Of the 24 pneumothorax cases, all were isolated, presented as minimal pneumothorax, and were managed with oxygen inhalation treatment. Similarly, the remaining 24 hemothorax cases were isolated, measured in millimeters, reported in thorax computed tomography reports, spontaneously resorbed, and did not require surgical intervention. None of the cases in this dataset necessitated further surgical interventions such as emergent thoracotomy or elective surgical procedures.

**Table 2:** Complications and interventions

	Number of patients	Number of performed tube thoracostomy
Pneumothorax	39	15 (38.4%)
Hemopneumothorax	21	21 (100%)
Hemothorax	27	3 (11.1%)
Contusion	22	
Flail chest	7	
Mechanical ventilator support	4	

The number of the male patients was significantly higher (p=0.000). However, there was no significant difference between gender and complications (p=0.402). Motor vehicle accidents caused complications significantly more often than other mechanisms of trauma (p=0.002).

The mean value of the number of fractured ribs in Group 2 was significantly higher compared to that in Group 1 (4.17 vs. 1.85; p=0.000). Additionally, complication rates in Group 2 (n=82) were significantly higher (p=0.000). These two groups were also assessed based on the mean age. The data in the age category were normally distributed. Therefore, an independent t test was performed, revealing that Group 2 had a significantly higher mean age (56.24 vs. 51.08; p=0.033).

To analyze the impact of variables in this database on the ISS [6] and CTS [7] scoring systems, multiple linear regression analysis was performed to distinguish which variables in this database affect these scoring systems (Table 3). The same variables were compared.

**Table 3:** Scoring systems divided by groups related to complications

	Injury Severity Score		Chest Trauma Score	
Group	Complication (-)	Complication (+)	Complication (-)	Complication (+)
N	98	76	98	76
Mean	7.31	14.67	2.92	3.45
Minimum	6	11	2	2
Maximum	19	41	4	7
Test/p	Mann-Whitney U test u: 674,00 p: 0,000		Independent t test t: -3,52 p: 0,001	

Variables were analyzed for their effect on ISS (Table 4). Age, three or more fractured ribs, and the mechanism of trauma (motor vehicle injuries) had an effect on ISS. Age had a negative effect on ISS, indicating that ISS was higher in the younger population, not the older.

**Table 4:** Linear regression analysis, covariates affect Injury Severity Score

	Standardized coefficient Beta	p-value
Age	-0.206	0.003
Gender	-0.071	0.290
Number of fractures (<3. ≥ 3)	0.489	0.000
Mechanism of trauma	0.198	0.003
Bilateral fractures	0.006	0.929

The independent covariates that affected CTS were lined up by effect: age > bilateral fractures > three or more fractured ribs > mechanism of trauma (p<0.05 in all covariates). This analysis showed that CTS was higher in the older population, contrasting with ISS (Table 5). In this study, the mortality rate was 0%.

**Table 5:** Linear regression analysis, covariates affect Chest Trauma Score

	Standardized coefficient Beta	p-value
Age	0.667	0.000
Gender	-0.055	0.103
Number of fractures (<3. ≥ 3)	0.016	0.001
Mechanism of trauma	0.073	0.029
Bilateral fractures	0.491	0.000

To explore the correlation between pulmonary complication rates and covariates, a binary logarithmic regression test was employed. Univariable tests indicated significant correlations between pulmonary complication status and the mechanism of trauma, the number of fractured ribs category, and bilateral cases. However, the multivariable results revealed that, in this study, only the mechanism of trauma and the number of fractured ribs categories maintained a significant correlation (Table 6).

**Table 6:** Binary logarithmic regression analysis, covariates correlated with pulmonary complications

	Univariable			Multivariable		
	OR	95%Cl	p-value	aOR	95%Cl	p-value
Age	1.00	0.98-1.02	0.700	0.99	0.97-1,01	0.561
Bilateral fractures	18.18	2.3-143.3	0.006	6.98	0.73-66.77	0.091
Three or more fractured ribs	9.93	4.92-20.03	0.000	9.65	4.44-20.98	0.000
Motor vehicle trauma	2.81	1.45-5.45	0.002	0.36	0.16-0.81	0.014
Gender	0.73	0.36-1.50	0.403	0.95	0.38-2.37	0.919

## Discussion

The chest cavity was divided into upper, middle, and lower parts, each consisting of the same number of ribs. The middle part covered the most significantly affected ribs. This can be explained by the fact that the middle part covers the broadest surface of the chest cavity and the longest ribs anatomically.

In many studies, motor vehicle accidents are the most common mechanism of trauma [2]. These accidents, categorized as high-energy traumas, usually cause multisystemic traumas. In this study, 17 cases had multisystemic traumas, mostly caused by traffic accidents. Ziegler et al.'s study similarly identified motor vehicle crashes as the most common mechanism [2]. Kaplan states that ISS is significantly lower in cases with a used seatbelt [8]. In this study, "fall from >2 meters high" was the most common mechanism, although the mechanism most correlated with complication rates was motor vehicle traumas.

In the study of Bergeron et al., a fall from height was common in the elderly population, while motor vehicle accidents were more common in the younger population [4]. Likewise, in the current study, a fall from height was the most common mechanism of trauma, particularly in elderly patients (n=78, mean age: 57). Motor vehicle accidents followed it with a younger population (n=54, mean age: 50).

Many scoring systems use three or more rib fractures as a threshold value [6,7,9]. Chien et al. noted that the number of displaced rib fractures is more predictive of complications in chest trauma patients [10]. However, Lee et al. reported that patients with three or more rib fractures need a third-stage healthcare supply [11]. This study also revealed that patients with three or more rib fractures tend to have pulmonary complications.

Brasel et al. stated in their study that female gender is protective [5]. Bergeron et al. reported that male gender is related to a higher mortality rate [4]. In this study, gender had no effect on scoring systems and complication rates.

Brasel et al. reported increased odds of death in children zero to four years of age as well, which was significant in isolated thoracic trauma patients [5]. Sırmalı et al. reported that children’s ribs are more flexible by nature compared to adults [12]. Hence, the consequences of thoracic trauma in children may be different from that in adults. Therefore, the number of fractured ribs may be a misleading indicator in children of the severity of the trauma [12]. The current study's database included patients aged 22-89, so there was no pediatric chest trauma case in the study.

Studies show that pneumonia is a risk factor correlated with increased mortality rates [4]. In the study of Chapman et al., pneumonia in non-ventilated patients was defined as two or more of the following: productive cough, temperature greater than 38.4 Celsius, leukocytosis of greater than 11,000 per mm^3^, and new or worsening chest x-ray infiltrate [13]. In the present study, we didn’t have a case that matched those criteria.

Although the current study reflects the profile of blunt chest trauma patients and the medical practice of a thoracic surgeon working alone at a healthcare center in the periphery, there are some limitations such as retrospective nature, absence of patients who underwent emergent or elective surgical interventions or who had pneumonia, absence of pediatric cases, and the precise nature of the fractures (displacement or fragmentation). Multicenter and prospective studies are needed to evaluate comprehensively.

## Conclusions

Considering the high morbidity and mortality rates associated with multiple rib fractures and unstable chest wall injuries, identifying patients requiring close monitoring is of utmost importance. In the current study, three or more rib fractures, bilateral rib fractures, and high-energy traumas were observed to be significant risk factors, particularly in the elderly population. We believe that hospitalization and careful observation are appropriate for patients meeting these criteria. Additionally, we recommend hospitalization and follow-up for the management of pain and complications, as well as the treatment of lung function impairments, for patients meeting these criteria.
